# The value of routine chest radiographs after minimally invasive cardiac surgery: an observational cohort study

**DOI:** 10.1186/s13019-014-0174-9

**Published:** 2014-11-11

**Authors:** Martijn Tolsma, Mohamed Bentala, Peter M J Rosseel, Bastiaan M Gerritse, Homme A J Dijkstra, Paul G H Mulder, Nardo J M van der Meer

**Affiliations:** Department of Anesthesiology & Intensive Care, Isala Klinieken, Dokter van Heesweg 2, Zwolle, 8025 AB The Netherlands; Department of Cardiothoracic Surgery, Amphia Hospital, Molengracht 21, Breda, 4818 CK The Netherlands; Department of Anesthesiology & Intensive Care, Amphia Hospital, Molengracht 21, Breda, 4818 CK The Netherlands; Department of Radiology, Amphia Hospital, Molengracht 21, Breda, 4818 CK The Netherlands; Amphia Hospital, Amphia Academy, Molengracht 21, Breda, 4818 CK The Netherlands; TiasNimbas Business School, Tilburg University, Warandelaan 2, Tilburg, 5037 AB The Netherlands

**Keywords:** Chest radiographs, Cardiac surgery, Intensive care unit

## Abstract

**Background:**

Chest radiographs (CXRs) are obtained frequently in postoperative cardiac surgery patients. The diagnostic and therapeutic efficacy of routine CXRs is known to be low and the discussion regarding the safety of abandoning these CXRs after cardiac surgery is still ongoing. We investigated the value of routine CXRs directly after minimally invasive cardiac surgery.

**Methods:**

We prospectively included all patients who underwent minimally invasive cardiac surgery by port access, ministernotomy or bilateral video-assisted thoracoscopy (VATS) in the year 2012. A direct postoperative CXR was performed on all patients at ICU arrival. All CXR findings were noted, including whether they led to an intervention or not. The results were compared to the postoperative CXR results in patients who underwent conventional cardiac surgery by full median sternotomy over the same period.

**Main results:**

A total of 249 consecutive patients were included. Most of these patients underwent valve surgery, rhythm surgery or a combination of both. The diagnostic efficacy for minor findings was highest in the port access and bilateral VATS groups (56% and 63% versus 28% and 45%) (p < 0.005). The diagnostic efficacy for major findings was also higher in these groups (8.9% and 11% versus 4.3% and 3.8%) (p = 0.010). The need for an intervention was most common after minimally invasive surgery by port access, although this difference was not statistically significant (p = 0.056).

**Conclusions:**

The diagnostic efficacy of routine CXRs performed after minimally invasive cardiac surgery by port access or bilateral VATS is higher than the efficacy of CXRs performed after conventional cardiac surgery. A routine CXR after these procedures should still be considered.

## Background

Chest radiographs (CXRs) are obtained frequently for intensive care unit (ICU) patients, on a routine basis, after a change in clinical situation or after surgery and other certain procedures. Multiple investigators have studied the clinical value of routine CXRs following central venous catheterization, endotracheal intubation and chest tube placement or removal [1]-[12]. Others have studied the value of daily routine CXRs in a mixed ICU population or in mechanically ventilated patients only [13]-[22] The diagnostic and therapeutic efficacy of these routine CXRs is known to be low [1]-[3],[6]-[9],[11],[13],[14],[16]-[19],[22]. Investigators comparing a routine CXR strategy with an on-demand CXR strategy were not able to show any difference in outcome measures [23]-[29], but a more recent meta-analysis by Ganapathy et al. indicated that study populations were small and that eventually missed findings in a restrictive strategy were not evaluated frequently enough [27]. Moreover, the discussion regarding specific indications of CXRs in critically ill patients and the safety of abandoning routine CXRs is still ongoing [25]-[27].

In accordance with the results of general studies on this topic, the clinical value of routine chest radiographs after cardiac surgery is reported to be low [30]-[34]. Abandoning routine CXRs in this population may only be safe when patients at risk are identified and certain indications of CXRs are stated. Minimally invasive cardiac surgery patients represent a population that might benefit from routine CXRs after surgery. Minimally invasive cardiac surgery has become increasingly popular over the past decade and is currently safe and effective [35]-[37]. Surgical access is obtained by (antero)lateral thoracotomy (port access), video-assisted thoracoscopy (VATS), mini-sternotomy or a parasternal approach. The procedures involved concern mainly valve surgery and rhythm surgery. The aims of minimally invasive surgery are to reduce blood loss, the number of reoperations, postoperative pain and the length of ICU stay and to promote a quick recovery and provide a cosmetically better result [35]-[38]. To our knowledge, there are no reports on CXR findings after minimally invasive cardiac surgery. Hypothetically, there are some findings that can be diagnosed by a postoperative CXR. These results might be related to the place of surgical access (pneumothorax, subcutaneous emphysema), temporary one lung ventilation technique (atelectasis), less surgical field visualization and hemostasis (haemothorax) or the need for invasive device placement (pulmonary artery catheter, temporary transvenous pacing wire). We performed a study on the efficacy of CXRs obtained directly after minimally invasive cardiac surgery.

## Methods

This prospective, observational, single-center study was performed on a tertiary 24-bed closed format ICU, admitting medical, surgical and cardiothoracic surgical patients. The medical staff consisted of 12 intensivists and 8 residents in ICU medicine. The study protocol was approved by the local ethics committee of the Amphia Hospital (AMOA; Adviescommissie Mensgebonden Onderzoek Amphia, mr. F. de Haan). This is the hopsital where the study was conducted. The need for informed consent was waived because no interventions were applied to the patients apart from the common and current local practice. All patient data were obtained anonymously.

The study population was a part of another prospective study on CXR findings in all cardiosurgical patients admitted in the year 2012. We selected all consecutive patients who underwent minimally invasive cardiac surgery during this year, concerning patients for valve surgery, rhythm surgery or a combination of both. The patients were divided by the type of surgical access; port access, mini-sternotomy or bilateral VATS. All patients who underwent cardiac surgery by conventional full median sternotomy over the same period were used as a control group. Patients were admitted to the ICU directly after surgery. For all minimally invasive surgery patients, a CXR was obtained routinely at ICU arrival. For patients who underwent conventional surgery, a CXR was performed on-demand postoperative or routinely on the morning of the first postoperative day.

Demographic data and surgery characteristics were collected for all patients. The mean age and the median duration of ICU stay were calculated. All CXRs were assessed both by a radiologist and an ICU physician. CXR findings were classified according to the overview presented in Table 1 and were divided into minor findings and major findings. Only new findings were incorporated into analysis, and abnormalities already present on a preoperative CXR were not taken into consideration again.

**Table 1:** **Classification of radiologic findings**

Minor findings^1^	Major findings^2^
Minimal pleural effusion	Severe pleural effusion
Small atelectasis	Large atelectasis
Minimal pulmonary congestion	Severe pulmonary congestion
Small consolidation	Large consolidation
	Malposition of invasive devices
	Widened mediastinum
	Large subcutaneous emphysema
	Haemothorax
	Pneumothorax
	Pneumomediastinum
	Pneumopericardium
	Free air under diaphragm

^1^Involvement of less than one lobe, and/or judged `normal postoperative’.

^2^Involvement of one lobe or more, and/or judged `no normal postoperative finding’.

All CXR abnormalities were noted. For major abnormalities it was also noted whether this abnormality led to an intervention. Possible interventions were chest tube placement, reposition of invasive devices, diuretic therapy, echocardiographic assessment and re-operation. The proportion of CXRs that showed minor and major findings was calculated, as was the proportion of CXRs with findings that led to a subsequent intervention. The diagnostic efficacy (the number of abnormalities divided by the total number of CXRs) and therapeutic efficacy (the number of interventions based on CXR abnormalities divided by the total number of CXRs) were also calculated. Finally, the CXR results of minimally invasive cardiac surgery patients were compared to the postoperative CXR results for patients who underwent cardiac surgery by conventional median sternotomy in the same period.

Data analysis was performed using IBM SPSS Statistics v21.0 for Windows. Differences in the percentages of findings and interventions were tested using Fisher’s exact test. Other differences were tested using a two sample t-test or a Mann Whitney test where appropriate. A p-value below 0.05 was used to denote significance.

## Results

Table 2 shows the baseline characteristics of the study population. A total of 249 consecutive patients who underwent minimally invasive cardiac surgery by port access (n = 124), mini-sternotomy (n = 69) or bilateral VATS (n = 56) were included. Most of these patients underwent valve surgery, rhythm surgery or a combination of both. Their CXR results were compared to the CXR results of 1102 patients who underwent conventional cardiac surgery in the same period. The most frequent procedure in this population was coronary artery bypass grafting (CABG) eventually combined with valve surgery or rhythm surgery. Patients who had cardiac surgery by port access or mini-sternotomy were less frequently male (55% and 49% compared to 73%) (p < 0.005). Patients in the bilateral VATS group were younger (61 ± 8 years compared to 69 ± 9 years) (p < 0.005). The mean length of ICU stay was shorter for all minimally invasive surgery groups when compared to that of the conventional cardiac surgery group (1.6, 1.5 and 1.1 days compared to 2.0 days) (p = 0.007).

**Table 2:** **Baseline data of the study population and procedures, divided by type of surgical access**

	PA	MS	BV	CS	p
Patients, n	124	69	56	1102	
Gender, male, n (%)	68 (55)	34 (49)	41 (73)	809 (73)	<0.005
Age, years, mean ± SD	68 ± 10	69 ± 12	61 ± 8	69 ± 9	<0.005
Length of ICU stay, days, mean	1.6 (1–9)	1.5 (1–20)	1.1 (1–4)	2.0 (1–66)	0.007
(range)					
Length of ICU stay 1 day, n (%)	93 (75)	63 (91)	53 (95)	847 (77)	<0.005
Procedures; n (%)					
CABG	-	-	-	655 (49)	<0.005
CABG with valve surgery	-	-	-	177 (13)	<0.005
CABG with rhythm surgery	-	-	-	21 (2)	0.345
Valve surgery	78 (63)	69 (100)		140 (13)	<0.005
Valve surgery with aortic surgery	-	-	-	42 (4)	0.016
Valve surgery and rhythm surgery	43 (35)	-	-	32 (3)	<0.005
Aortic surgery	-	-	-	24 (2)	0.230
Rhythm surgery	-	-	56 (100)	-	<0.005
Other surgery	3 (2,4)	-	-	11 (1)	0.389

PA = Port Access; MS = Mini-sternotomy; BV = Bilateral Video Assisted Thoracoscopy; CS = Conventional Sternotomy; n = Number; SD = Standard Deviation; ICU = Intensive Care Unit; IQR = Interquartile Range; CABG = Coronary Artery Bypass Graft; VATS = Video Assisted Thoracoscopy.

Table 3 shows a comparison of the diagnostic and therapeutic efficacies for CXRs performed after the different types of minimally invasive cardiac surgery and CXRs performed after conventional cardiac surgery. The diagnostic efficacy for minor findings was highest in the port access and bilateral VATS groups (56% and 63% compared to 28% and 45% in the mini-sternotomy and conventional surgery groups) (p < 0.005). The diagnostic efficacy for major findings was also higher in the port access and bilateral VATS groups (8.9% and 11% compared to 4.3% and 3.8%) (p = 0.010). The need for an intervention was most common after minimally invasive surgery by port access (4.8% of cases compared to 1.5% of cases after conventional surgery), although this difference was not statistically significant (p = 0.056).

**Table 3:** **Comparison of diagnostic and therapeutic CXR values between different types of surgery**

	PA	MS	BV	CS	p
	(n = 124)	(n = 69)	(n = 56)	(n = 1102)	
CXRs with any finding, n (%)	80 (65)	22 (32)	41 (73)	540 (49)	<0.005
CXRs with minor findings only, n (%)^1^	79 (56)	19 (28)	35 (63)	498 (45)	<0.005
CXRs with major findings, n (%)^1^	11 (8.9)	3 (4.3)	6 (11)	42 (3.8)	0.010
CXRs with subsequent intervention, n (%)^2^	6 (4.8)	0 (0)	0 (0)	17 (1.5)	0.056

CXR = Chest Radiograph; PA = Port Access; MS = Mini-sternotomy; BV = Bilateral Video Assisted Thoracoscopy; CS = Conventional Sternotomy; n = Number.

^1^Diagnostic efficacy.

^2^Therapeutic efficacy.

An overview of minor postoperative CXR findings is shown in Table 4. Pleural effusion, atelectasis and consolidation were observed more frequent after minimally invasive surgery by port access and bilateral VATS (p = 0.019, p < 0.005 and p < 0.005), whereas pleural effusion and atelectasis were observed less frequently in the mini-sternotomy group. Minor pulmonary congestion was observed significantly more frequently in the bilateral VATS group (p < 0.005).

**Table 4:** **Minor CXR findings**

	PA	MS	BV	CS	p
	(n = 124)	(n = 69)	(n = 56)	(n = 1102)	
Finding; n (%)					
Pleural effusion	22 (18)	3 (4.3)	12 (21)	171 (16)	0.019
Atelectasis	36 (29)	6 (8.7)	22 (39)	257 (23)	<0.005
Pulmonary congestion	12 (9.7)	11 (14)	19 (34)	173 (16)	<0.005
Consolidation	19 (15)	4 (5.8)	10 (18)	63 (5.7)	<0.005

PA = Port Access; MS = Mini-sternotomy; BV = Bilateral Video Assisted Thoracoscopy; CS = Conventional Sternotomy; CXR = Chest Radiograph; n = Number.

The major findings are presented in Table 5. The values shown are small, and only severe pulmonary congestion, large consolidation and large subcutaneous emphysema were observed statistically more frequently in the port access or bilateral VATS groups (p = 0.013, p = 0.024 and p = 0.016). A pneumothorax, a haemothorax and malposition of invasive devices were also observed more frequently in all minimally invasive surgery groups, although this finding was not significant.

**Table 5:** **Major CXR findings**

	PA	MS	BV	CS	p
	(n = 124)	(n = 69)’	(n = 56)	(n = 1102)	
Finding; n					
Large pleural effusion/haemothorax	2 (1.6)	1 (1.4)	0 (0)	5 (0.5)	0.204
Large atelectasis	0 (0)	0 (0)	0 (0)	2 (0.2)	1.000
Severe pulmonary congestion	2 (1.6)	0 (0)	1 (1.8)	1 (0.1)	0.013
Large consolidation	2 (1.6)	0 (0)	0 (0)	0 (0)	0.024
Malposition invasive devices	3 (2.4)	2 (2.9)	1 (1.8)	10 (0.9)	0.104
Widened mediastinum	1 (0.8)	0 (0)	2 (3.6)	13 (1.2)	0.299
Large subcutaneous emphysema	1 (0.8)	0 (0)	1 (1.8)	0 (0)	0.016
Pneumothorax	3 (2.4)	2 (2.9)	1 (1.8)	10 (0.9)	0.104
Pneumopericardium	0 (0)	0 (0)	0 (0)	2 (0,2)	1.000

PA = Port Access; MS = Mini-sternotomy; BV = Bilateral Video Assisted Thoracoscopy; CS = Conventional Sternotomy; CXR = Chest Radiograph; n = Number.

## Discussion

We observed that routine CXRs obtained after minimally invasive cardiac surgery by port access or bilateral VATS have a higher diagnostic value than CXRs performed after cardiac surgery by mini-sternotomy or conventional full median sternotomy. The high diagnostic efficacy for minor findings in all groups (40-60%) is comparable to the results reported in previous studies for cardiac surgery patients and studies performed in a general ICU population. [22],[33] We observed diagnostic efficacies of 8.9% and 11% for major findings after minimal invasive cardiac surgery by port access and bilateral VATS, which is clearly higher than what has been observed in more recent studies on the efficacy of chest radiographs after conventional cardiac surgery or for critically ill patients in generally [18],[31],[33]. A low therapeutic efficacy (1% to 4%) does correspond with previous findings [16],[18],[33]. We only observed a higher therapeutic value for CXRs after cardiac surgery by port access (4.8%).

The difference between patients who underwent minimally invasive cardiac surgery by port access or bilateral VATS and other cardiosurgical patients, as mentioned above, most likely be related to the complications of these surgical procedures. We were able to confirm a more frequent presence of atelectasis following a one lung ventilation technique. In addition, although not statistically significant, we did observe the relatively frequent presence of a pneumothorax, haemothorax and malposition of invasive devices after minimally invasive procedures. These results may be related to the place of surgical access, difficult hemostasis and the need for invasive device placement.

Because the discussion regarding the indications of CXRs in ICU patients and the specific clinical situations in which routine CXRs should still be performed is still ongoing, our results may be of interest. In our opinion, and in agreement with our findings, there is still a place for routine CXRs directly after minimally invasive cardiac surgery by port access or bilateral VATS. This is in contradiction to patients after uncomplicated conventional cardiac surgery or minimally invasive surgery by mini-sternotomy.

Our study is limited by the fact that it was a single-center study and that it was performed according to a routine CXR strategy protocol. A postoperative CXR was performed anyway for every patient. The study is also limited by the fact that we used an observational cohort study design without randomization or blinding. On the other hand, according to our design, no findings could be missed and the frequency of eventual subsequent interventions was evaluated.

## Conclusion

Routine CXRs performed after minimally invasive cardiac surgery by port access or bilateral VATS have a higher diagnostic efficacy than CXRs performed after cardiac surgery by mini-sternotomy or conventional full median sternotomy. A routine CXR after these procedures should still be considered.

## Abbreviations

CXR: Chest radiograph
ICU: Intensive care unit
VATS: Video assisted thoracoscopy
IBM: International business machines
SPSS: Statistical package for the social sciences
CABG: Coronary artery bypass grafting
